# Age-Dependent and Tissue-Specific Alterations in the rDNA Clusters of the *Panax ginseng* C. A. Meyer Cultivated Cell Lines

**DOI:** 10.3390/biom10101410

**Published:** 2020-10-06

**Authors:** Galina N. Chelomina, Konstantin V. Rozhkovan, Olga L. Burundukova, Tatiana Y. Gorpenchenko, Yulia A. Khrolenko, Yuri N. Zhuravlev

**Affiliations:** 1 Federal Scientific Center of the East Asia Terrestrial Biodiversity, Far-Eastern Branch of Russian Academy of Science, Vladivostok 690022, Russia; 27.tomcat@gmail.com (K.V.R.); burundukova.olga@gmail.com (O.L.B.); gorpenchenko@biosil.ru (T.Y.G.); khrolenko@biosoil.ru (Y.A.K.); yuzhuravlev@biosoil.ru (Y.N.Z.); 2 Saint-Petersburg State University Clinic, St. Petersburg 190103, Russia

**Keywords:** *Panax ginseng*, 18S rDNA, rRNA secondary structure, cell lines, polyploidy, somaclonal variation, ribosomopathies

## Abstract

Long-term cultivation of *Panax ginseng* cell lines leads to a decreasing synthesis of the biologically active substances used in traditional medicine. To gain insight into the cellular mechanisms which may influence this process, we analyzed variations within the rDNA cluster of the Oriental ginseng cell lines. The cell lines were cultivated for 6 and 24 years; the number of nucleoli and chromosomes was analyzed. The complete 18S rDNA sequences were cloned and sequenced. The nucleotide polymorphism and phylogenetic relations of the sequences were analyzed, and the secondary structures for separate 18S rRNA regions were modeled. The 18S rDNA accumulated mutations during cell cultivation that correlate well with an increase in the number of chromosomes and nucleoli. The patterns of nucleotide diversity are culture-specific and the increasing polymorphism associates with cytosine methylation sites. The secondary structures of some 18S rRNA regions and their interaction can alter during cultivation. The phylogenetic tree topologies are particular for each cell line.The observed alterations in rDNA clusters are associated with a somaclonal variation, leading to changes in the pattern of intracellular synthesis during cell cultivation. The identified divergent rRNAs could provide additional gene expression regulation in *P. ginseng* cells by forming heterogeneous ribosomes.

## 1. Introduction

*Panax ginseng* Meyer is one of the most valuable herbs used in Chinese traditional medicine. However, the uncontrolled harvesting of wild ginseng roots has led to an endangered status of this unique plant, so one of the important parts of its conservation strategy is the cultivation of *P. ginseng* cells in vitro. Plant cell cultures are a promising source for the large-scale and cost-effective production of high-purity valuable secondary metabolites. Plant cell lines have attracted scientists’ attention due to their ability to produce biologically active substances with strong therapeutic effects against various diseases and aging [1,2,3,4,5], and ginseng cell lines are proposed to be a valuable source of the unique biologically active saponins called ginsenosides. Unfortunately, cell lines often lose their ability to produce biologically active substances during long-term cultivation, and there are very few successful examples of their utilization for the commercial production of secondary metabolites (e.g., shikonin and paclitaxel) [1,2,3,4]. The true reasons for this instability are still unknown, and should be clarified for the successful exploitation of plant cell lines for the purposes requiring long-term cultivation. Additionally, cell cultures are appropriate systems for studying the regulation of metabolic pathways and the involved genes [4].

It has been demonstrated that the in vitro culture of plant tissue induces various mutations and changes in epigenetic marks, and genetic variation has been observed both in cultured cells and in plants regenerated from the cultured cells. These mutations include cytological abnormalities such as ploidy changes and chromosome rearrangements, single-base substitutions, changes in the copy number of repeated sequences, and alterations in DNA methylation patterns [6,7,8]. Such mutations in plant cell cultures are referred to as somaclonal variation, which has been described for undifferentiated cells, isolated protoplasts, calli, tissues, as well as in many traits, including plant height and biomass, grain yield, disease and insect resistance, tolerance to acidic and saline soils, and agronomic performance [8,9,10,11,12,13]. One of the possible reasons for the somaclonal variation observed in *P. ginseng* cell cultures is the decrease in genomic stability, including polyploidy, aneuploidy, and multi-nuclearization [14]. Another reason may be associated with the accumulation of point mutations within the genome of the cell line, which has been described for a ginseng housekeeping gene (*actin*), genes participating in ginsenoside biosynthesis (*PAL* and *DDS*), and a development-regulating gene (*SERK*) [15].

The rRNA gene for small ribosomal subunit (SSU) is a part of the rDNA cistron, which consists of three coding (18S-SSU, 5.8S, and 28S-LSU) and four spacer regions (internal transcribed spacers (ITS1 and ITS2), external transcribed spacer (ETS), and intergenic spacer (IGS)). To satisfy the transcriptional requirements, a high copy number of the rDNA cistrons is needed for each organism, and mechanisms of the concerted evolution are directed to their homogenization within genomes and within species (among individuals). However, intraspecific and intragenomic sequence variability appear to be common characteristics for the rDNA sequences [16,17,18]. Previously, we also revealed the existence of 18S rDNA nucleotide variability in leaves of the cultivated *Panax ginseng* plant, which in theory may affect the production of the biologically active compounds [19].

The purpose of this study was to determine the patterns of intragenomic nucleotide polymorphism in the complete 18S rDNA sequences of *Panax ginseng* cell lines, which are different at the time of cultivation and in the tissue source. We focused our attention on the nucleotide polymorphism of the cell cultures in order to determine whether the existence of differences among 18S rDNA sequences could decrease the ginsenoside biosynthesis. We assessed the sequence polymorphism by cloning and sequencing multiple copies of the 18S rDNA, reconstructing phylogenetic relationships as well as modeling putative secondary structures for some regions of the 18S rRNA molecule. We also analyzed cytogenetic characteristics of genomic instability, including the number of chromosomes per cell and the number of nucleoli per nucleus.

## 2. Materials and Methods

### 2.1. Plant Material

The long-term cultivated cell lines *1s* and *1o* were established in 1988 at the Laboratory of Biotechnology (IBSS FEB RAS). Line *1s* was obtained from the stem of a 2-month-old plant of *Panax ginseng*, and line *1o* was obtained from the ovary of a wild-grown plant. The cell line *9s* was established in 2006 from the stem of a juvenile ginseng plant grown in an open experimental nursery. The analyzed *P. ginseng* cell lines have been passaged into fresh nutrient medium monthly for 6 (line *9s*) and 24 (lines *1s* and *1o*) years, totaling more than 70 and 280 passages, respectively. The cell line was cultivated at 24–25 °C in the dark on solid W medium [20] supplemented with 0.4 mg/L *p*-chlorophenoxyacetic acid (W4CPA) with a 30–35-day subculture interval.

### 2.2. Cytological Analysis

The cytological materials were prepared and analyzed as previously described [14]. Cell cultures (volume 0.5–1 mL) were pretreated in a 0.2% colchicine solution for 2 to 3 h at room temperature (about 22 °C), fixed in 3:1 ethanol:acetic acid mixture and stained with acetohematoxylin. For confocal observation of chromosomes and nuclei, DAPI (4′,6-diamidino-2-phenylindole, Molecular Probes Inc., Eugene, OR, USA) stain was done at the final concentration of 5 μg/mL. The slides were prepared using squash or drop techniques. The confocal microscopy investigation was done using equipment of the Instrumental Centre of Biotechnology and Gene Engineering of FSCEATB FEB RAS. The somatic chromosome number was studied in at least 60–100 well-spread metaphase plates. A 50% solution of silver nitrate was used to stain [21]. The numbers of nucleoli were counted on the same plates in 100–200 cells.

### 2.3. Isolation of Total DNA and Polymerase Chain Reaction Amplification

The isolation of total DNA was performed as described previously [22]. Amplification reactions were performed in 20 µL volumes containing 100 ng template DNA, 10 mM Tris-HCl (pH 8.5), 50 mM KCl, 2.5 mM MgCl_2_, 0.01% gelatin (*w*/*v*), 0.1 mM Triton X-100, 0.25 mM of each deoxynucleotide triphosphate, and 10 pM of each primer. We also used a mix (1:8) of *Pfu* and *Taq* DNA polymerases (Thermo Fisher Scientific, Waltham, USA) to minimize amplification errors. The polymerase chain reaction (PCR) primer sequences and temperature conditions were the same as those in [19].

### 2.4. Cloning and Sequencing of the 18S rDNA Sequences

The PCR products of two independent PCR reactions were separately cloned into the pTZ57R/T plasmid using the InsTAclone PCR product kit (Thermo Fisher Scientific, Waltham, USA). The PCR products were used for cloning procedures without any modification. Clones were generated according to the protocol recommended by the manufacturer and supplied with the cloning kit. The clones were amplified using M13 (‒20) (Syntol, Moscow, Russia) primers for colony screening and sequencing. The sequence of each clone was determined by an ABI 3130 Genetic Analyzer (Applied Biosystems, Foster City, CA, USA) using additional nested primers [23]. The nucleotide-sequence data of the 18S rRNA gene were recorded in GenBank nucleotide-sequence databases with the following accession numbers: KC593794‒KC593823.

### 2.5. Prediction of 18S rRNA Secondary Structure

A published model for the SSU rRNA secondary structure of wheat *Triticum aestivum* based on cryoelectron microscopy data [24] was used as a reference in our study. Secondary structures were predicted separately for each molecular segment and for each rRNA sequence and compared with those obtained for cultivated *Panax ginseng* plant [19]. Primary sequences were used as an input for the thermodynamically stable RNA secondary structure prediction in MFOLD software version 3.2 (http://mfold.rna.albany.edu), recommended for most RNA folding, using free-energy folding algorithms [25]. The expansion segments ES3b-c following the ES nomenclature of Gerbi (1996) and seven hairpins (h6, h7, h8, h17, h18, h27, and h33) of the small subunit rRNA were identified and modeled for each cell line. In most cases, the online algorithm suggested only one structure; structures are displayed using CorelDRAW X7.

### 2.6. Statistical Analysis and Phylogenetic Reconstructions

The Basic Local Alignment Search Tool program used for the alignments was performed using the MEGA 5 software package [26]. The distribution of nucleotide diversity (*π*) along the gene sequence (a “sliding-window” approach; window length of 100 sites, and step size of 25 sites) was analyzed using the DnaSP version 5.10 [27]. The intraspecific phylogenies for the data set of each cell line were reconstructed using a median-joining network [28] and minimum spanning trees (MST) generated in ARLEQUIN version 3.11 [29].

## 3. Results

### 3.1. Cytogenetic Analysis

**Figure 1:**
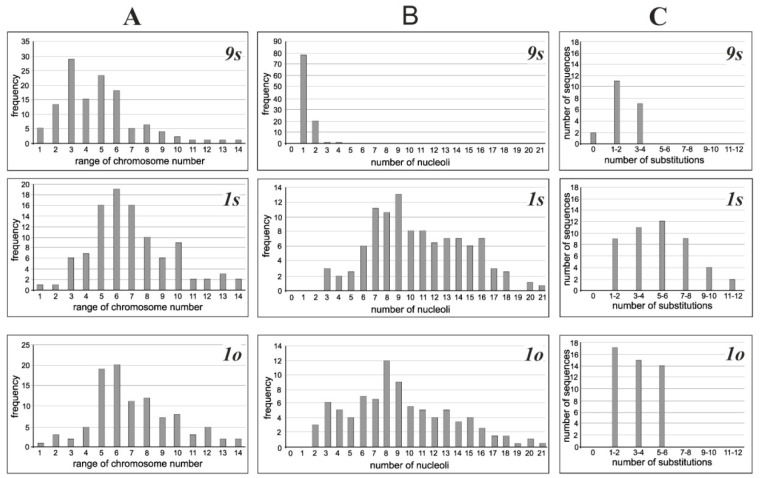
Distribution of chromosomes in ginseng cells, nucleoli in nucleus, and nucleotide substitutions in the 18S rDNA clones. (**A**) The number of chromosomes per cell, (**B**) the number of nucleoli per nucleus, and (**C**) the number of nucleotide substitutions per the 18S ribosomal DNA clone for different cell lines. Ranges of chromosome numbers—1: (1–10); 2: (11–20); 3: (21–30); 4: (31–40); 5: (41–50); 6: (51–60); 7: (61–70); 8: (71–80); 9: (81–90); 10: (91–100); 11: (101–110); 12: (111–120); 13: (121–130); 14: (>130).

The cytogenetic analysis found that in the cultivated *P. ginseng* cell lines the number of nucleoli in the interphase nuclei and chromosomes in cells varied significantly. The existence of mixoploidy in cell populations with different levels of variability as well as the presence of hyper-diploid and hypo-diploid, euploid, and aneuploid cytotypes was observed in all cell cultures. The highest level of chromosomal instability was characteristic of the long-term-cultivated (old) cell lines *1s* and *1o*, in which the number of chromosomes varied from 6 to 150, and hyper-diploid cells with 48–80 chromosome number constituting the modal class (over 50%) (Figure 1A). In short-term-cultivated (young) cell line *9s*, the range of variation in the number of chromosomes was 6–130 and the modal class (over 50%) constituted the cells chromosome number from 24 to 60 (Figure 1A).

**Figure 2:**
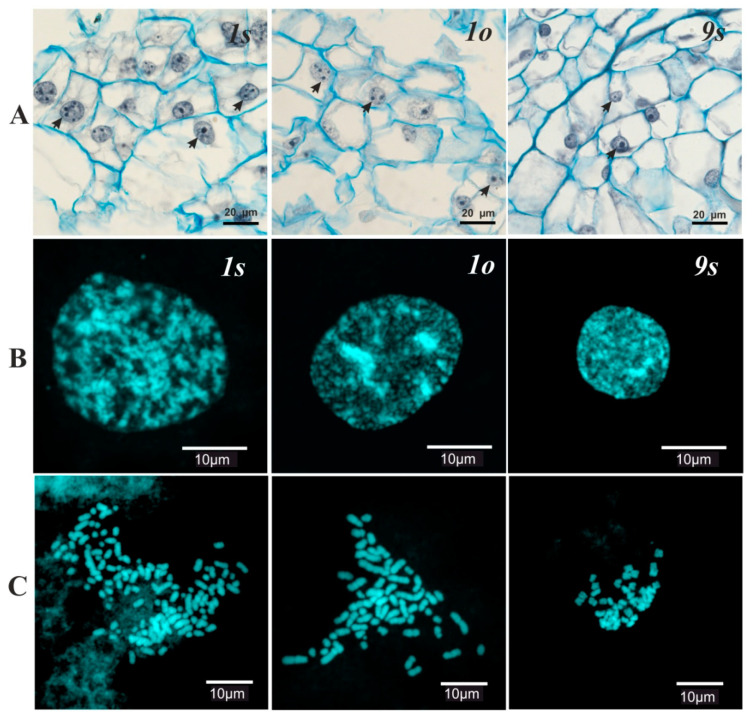
Cytogenetic variations in the *Panax ginseng* cell cultures. (**A**) Histology of the cell cultures (hematoxylin/alcian blue stained), arrows indicate the nucleoli. (**B**) The DAPI stained nuclei and (**C**) chromosomes.

The structure of the interphase nucleus also varied after the establishment of plants into in vitro culture (Figure 2). In the young cell line *9s*, the structure of the interphase nuclei did not differ from that of differentiated leaf tissue [19], while significant differences were observed in long-term-cultivated lines *1s* and *1o* (Table 1). In the nuclei of the old lines, a significant increase in the number of nucleoli and the appearance of out-of-the nucleolus genetic material (i.e., micro nucleoli with a size of less than 2 mm) were observed. The total number of nucleoli varied from 1 to 4 in the young culture and from 2 to 21 in old cultures (Figure 1B). The ratio of the average number of macro/micro nucleoli in the nucleus of cell populations in the *1s* and *1o* lines was 5.6/4.3 and 6.3/5.5, respectively (Table 1). Comparison of the interphase nuclei characteristics and the chromosome number data revealed a significant positive correlation (*r* = 0.98, *p* = 0.05) between the mean number of nucleoli in the nucleus and the average number of chromosomes in the cell.

### 3.2. Nucleotide Polymorphisms of the 18S rDNA Sequences in Cultivated Cell Lines

Of the 20 18S rDNA clones originated from the short-term-cultivated cell line *9s*, 90% were found to have 1–2 (55%) and 3–4 (35%) nucleotide differences (Figure 1C), while 10% were similar to the 18S rDNA sequence of *P. ginseng* plant from GenBank (accession number D83275). The clones of the long-term-cultivated cell lines had no identical sequences to the 18S rDNA of the *P. ginseng* plant from GenBank. In the *1o* cell line, the majority of clones (83%) differed in 1–2 and 3–4 nucleotides. In the *1s* cell line, the number of such clones was significantly lower (52%), however, only line *1s* presented clones with differences in 7–10 and more than 10 nucleotides. The 18S rDNA clones with 5–6 nucleotide substitutions were identified in both long-term-cultivated cultures (Figure 1C).

Most nucleotide substitutions in all the 18S rDNA sequences examined were transitions, and the *ts*/*tv* ratio was estimated to range from 9.3 in the short-term-cultivated cell line to 2.6 in long-term ones (Table 2). The short-term-cultivated cell line was found to be characterized by the presence of only three transversion types of substitutions, while the long-term-cultivated cell lines demonstrated the occurrence of all transversion types, with A→T being the most frequent nucleotide substitution (Table 2).

Almost half of the transitions (> 46%) in the short-term-cultivated cell line *9s* consisted of T→C substitutions. In each long-cultivated cell line, two different types of transitions were prevalent: A→G and C→T in *1s* (> 43%) and A→G and T→C in *1o* (> 47%) (Table 2). About 20% of nucleotide substitutions in *9s* and more than 30% of nucleotide substitutions both in *1s* and *1o* were associated with sites of cytosine methylation (Table 3). More than half of the revealed nucleotide substitutions in *9s* cell lines represented substitutions of T (NH_2_^−^) with other nucleotides (A-NH_2_^+^, C-NH_2_^+^, G-NH_2_^+^); we named them “DNA amination” substitutions. In the long-term-cultivated cell lines *1s* and *1o*, these mutations were less numerous (about 19–26%), as can be seen in Table 3.

The mean numbers of nucleotide substitutions per the 18S rRNA gene sequence (2.05–5.5), as well as those associated with sites of methylation (0.45–1.7), were significantly higher in the long-term-cultivated cell lines, particularly in *1s*. However, the number of amination substitutions was almost the same in the stem cell lines and slightly lower in the ovary cell line (about 1 and 0.9 substitutions per gene, respectively) (Table 3). In spite of pronounced variability in nucleotide sequences, the GC content in the 18S rDNA was stable in cell lines, demonstrating only minor differences (Table 3).

### 3.3. Distribution of Nucleotide Diversity along the 18S rDNA Sequences

**Figure 3:**
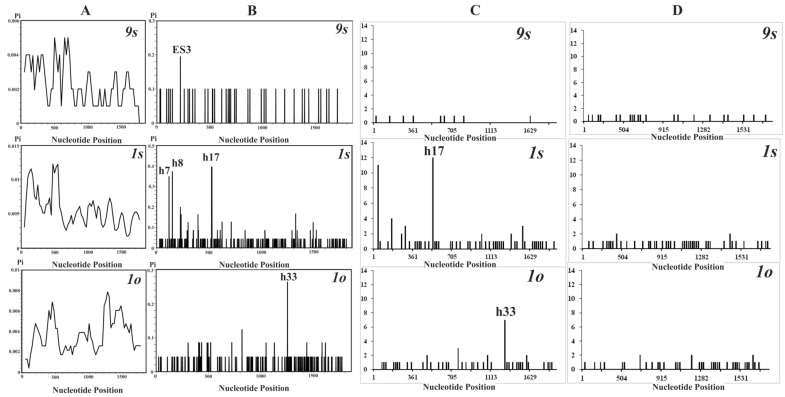
The 18S rDNA sequence variation in the *Panax ginseng* cell cultures. (**A**) Distribution patterns of all nucleotide substitutions along the gene as computed using the sliding-window option. Histograms for hotspots of variation for (**B**) all nucleotide substitutions, and for sites associated with (**C**) cytosine methylation and (**D**) DNA amination.

All rDNA alleles in the cell cultures were unique, with one exception (*9s*). The value of nucleotide diversity (*π*) for the cell lines *9s*, *1s*, and *1o* were estimated to be 0.00226, 0.00550, and 0.00360, respectively. The distribution patterns of nucleotide diversity, as well as hotspots of variation in the 18S rDNA sequences, were different for each cell line of *P. ginseng* (Figure 3A,B). In the cell cultures originating from vegetative stem tissue, the main peaks of nucleotide diversity were localized at the beginning of the gene sequences, while in the cultures originated from ovary the most pronounced peak was identified at the end part of gene sequences, within h33. In the short-term-cultivated stem cell line *9s*, a single small peak of nucleotide diversity was found within the ES3, while there were three main peaks in the long-term-cultivated stem line *1s* within h7, h8, and h17. 

For sites associated with methylation, hotspots of variation also revealed differences among *P. ginseng* cell lines (Figure 3C). In the long-term-cultivated cell lines, the main peaks of these substitution types localized in h17 of *1s* and h33 of *1o* coincided well with those for all nucleotide substitutions. In the short-term-cultivated culture, only the peaks resulting from the singleton substitutions were identified. With a few exceptions, the peaks associated with amination substitutions were caused by singleton nucleotide substitutions which were distributed almost evenly lengthwise along the gene sequences in all cell cultures, and no pronounced peaks were identified (Figure 3D).

### 3.4. Secondary Structures of the 18S rRNA ES3 and Helices

**Figure 4:**
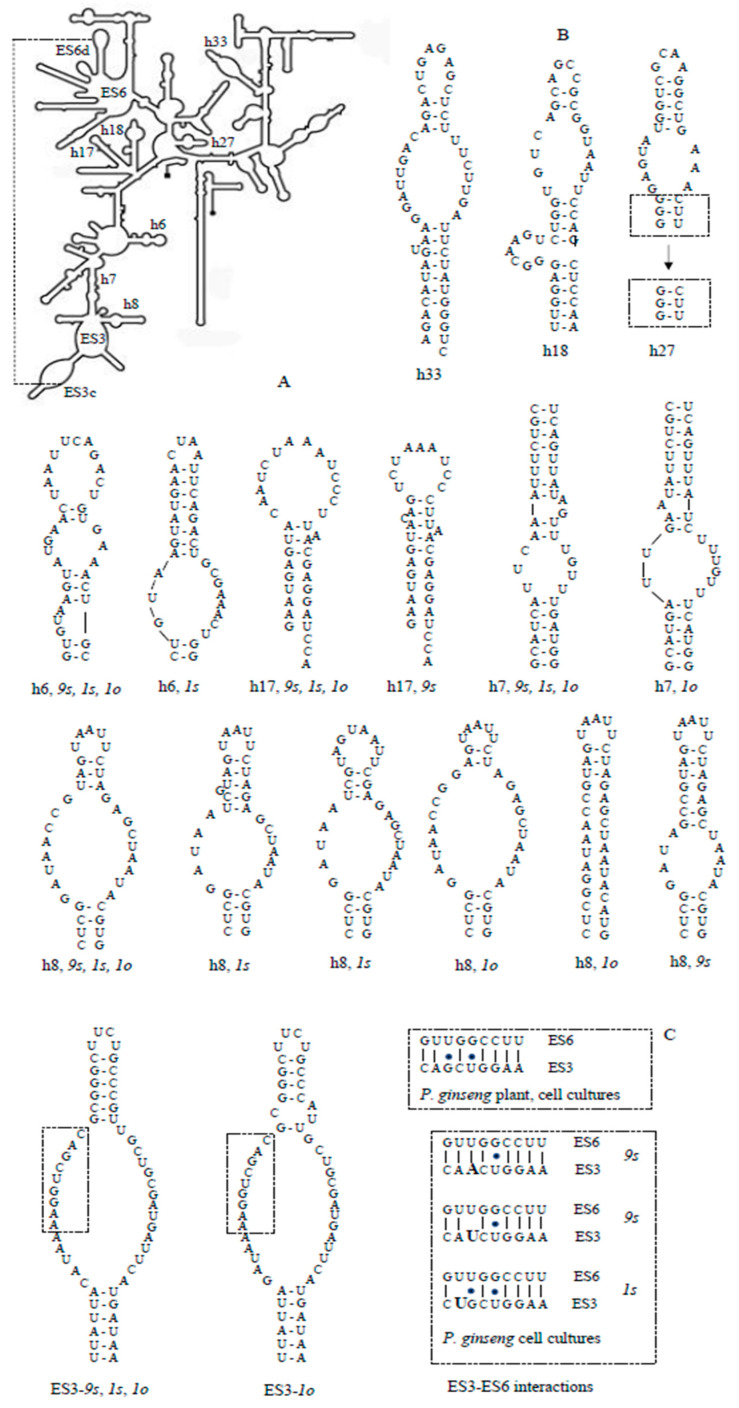
MFOLD prediction of the secondary structures for eight 18S rRNA regions of the *Panax ginseng* cell cultures. (**A**) Diagram of the secondary structure of wheat *Triticum aestivum* 18S rRNA (Armache et al., 2010) showing the model in this study region. The dotted line indicates the sites of interaction between ES3 and ES6; (**B**) The secondary structures of hairpins; (**C**) The secondary structures of expansion segment ES3b-c with sequences of the ES3–ES6 interaction sites.

The distribution pattern of nucleotide diversity along the 18S rDNA sequences (Figure 3) revealed an increased nucleotide polymorphism inside variable regions, both hairpins and ES3. Based on these data, we modeled the secondary structures of the SSU rRNA expansion segment ES3b-c, and hairpins h6, h7, h8, h17, and h33, as well as functionally important h18 and h27. The secondary structures for hairpins h18, h27, and h33 (Figure 4B) were estimated to be invariable in each cell line; at that, the structures of two first hairpins did not differ from those of wheat *Triticum aestivum* and *P. ginseng* plant [19]—that is, they were “canonical”. The h33 secondary structure of ginseng plant and cell lines was characterized by an almost symmetric interior loop which in wheat (with Cryo-EM analysis) was extremely asymmetric.

In the *9s* cell line, only two alternative structures to wheat were found, for h8 and h17 hairpins; however, canonical structures were formed by single alleles while alternative variants were common with those in *1s* and *1o*. In the h8 configuration, common for all ginseng cell lines and plants, the apical helix was shortened by half compared with the canonical structure, and the h17 configuration differed from the canonical one by an increased hairpin loop and an absence of one bulge loop (Figure 4).

In the *1s* cell line, the hairpins h6 (except for one allele variant) and h7 possessed canonical structures; the majority of h8 and all the h17 alleles had a common alternative specific hairpin structure. h6 and h8 formed one and two cell-line-specific alternative structures, respectively. The *1s* alternative variant of h6 is a singular configuration (among all cell lines), possessing only one interior loop and an increased apical helix. The first alternative configuration of h8 had a shortened apical helix with increased hairpin and interior loops, and the second one also had an additional one-nucleotide bulge loop (Figure 4b).

In the *1o* cell line, the only alternative structure was identified among the rDNA alleles as in h7 and ES3; all h17 alleles formed a ginseng-specific structure common with that of *1s* and *9s*, and h8 alleles had two line-specific alternative structures. The ES3 alternative structure differed with a small additional interior loop, and that of h7 had an increased interior loop and a decreased bulge loop. The first alternative configuration of h8 had a shortened apical helix with an increased interior loop, and the second one constituted only a hairpin loop with short helix followed by a vast region of non-pairing nucleotides (Figure 4B,C).

### 3.5. Phylogenetic Reconstructions for the 18S rDNA Gene Sequences

The total dataset of the 18S rRNA gene contained 113 sequences of length 1809 bp that resulted in 111 ribotypes, and no common ribotypes for cell cultures were identified which could be evidence of high specificity in a ginseng cell line. The values of both pairwise genetic *p*-distances (*D* = 0.002–0.005) and the average number of nucleotide substitutions (*K* = 4.095–9.937) for the 18S rRNA gene sequences differed among the cell lines, and were the highest in *1s* cell line. According to these indices, cell lines were closer to plant *P. ginseng* (*D* = 0.001–0.003) than to each other (*D* = 0.003–0.005) and the least variable line *9s* revealed the highest similarity with the cultivated plant.

**Figure 5:**
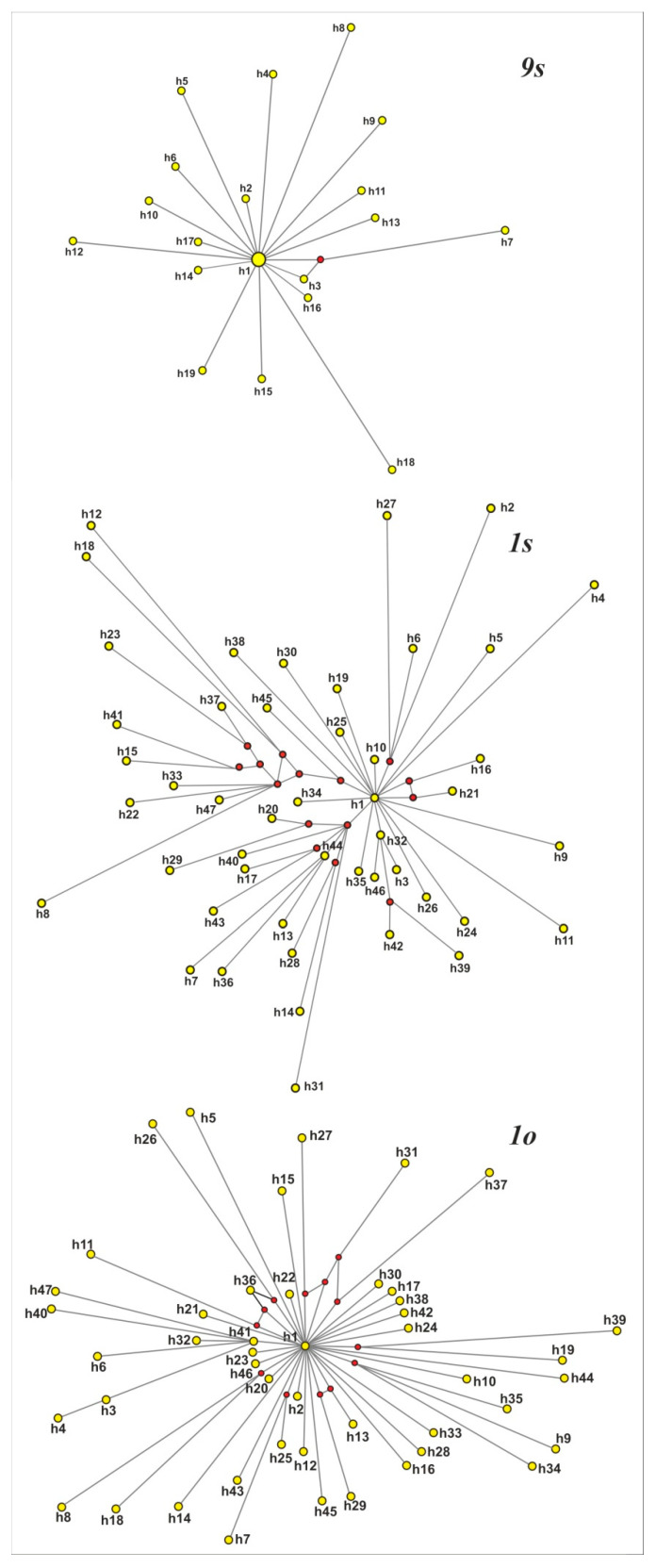
Phylogenetic relations of the 18S rDNA sequences in the *Panax ginseng* cell cultures.

The dataset for the young cell culture *9s* contained 19 alleles with 40 variable sites and 39 singleton mutations. The MJ network appeared as a star-like topology, suggesting a recent expansion from an ancestral sequence. The dataset for the old cell cultures contained 165 and 135 variable sites, of which 22 and 11 were parsimony-informative for *1s* and *1o*, respectively. The number of ribotypes for both old cell lines was 46. The MJ network for the *1s* cell line was relatively complex, comprising one major and a few secondary order star-like structures. The MJ network for the *1o* cell culture was more similar to that of *9s*, but rays in this reconstruction were much longer (Figure 5). For all cell cultures, MST trees (data are not shown) were quite similar to MJ reconstructions.

## 4. Discussion

To our best knowledge, the present study is the first attempt to describe the intragenomic variability of the 18S rDNA sequences during plant cell cultivation and find a possible connection between alterations in the ribosomal cluster and the ability of cells to synthesize secondary metabolites.

Genetic instability has different manifestations, including chromosome instability, clone heterogeneity, gene mutation, and amplifications, which are associated with genomic instability and both cell and organism aging [30,31,32]. Chromosomal instability of polyploid plant cells, which becomes apparent in chromosome variability, mixoploidy (polysomaty) of cell populations, and chimerism, is well known [6,7,8]. In this study, we revealed a significant increase in the range of the chromosome number variation with the prevalence of polyploid cells in both long-term-cultivated cell lines. However, the observed chromosomal variability was substantially lower than that of the 50–55-year-old ginseng cultures, in which the number of chromosomes ranged from 22 to 230 [33].

The increasing number of nucleoli in nuclei observed in this study is also well-known, and normal cells with different numbers of nucleoli have been reported to be different in specific activity to increase the protein synthesis [32,33]. Indeed, the nucleolus structure is dynamic in shape, size, and position within the nucleus. In addition to ribosome biogenesis, the nucleolus is involved in numerous other molecular functions (e.g., RNA processing, maturation, assembly, and export of RNA) and plays an important role in many cellular functions (e.g., regulation of the cell cycle, telomerase activity, aging, and stress responses) [34,35]. The impact of physical and chemical factors such as mutagens also causes an increase in the number of nucleoli in the nucleus of the plant, which is used in the environment as the nucleolus test for mutagenesis [33].

Many factors are known to affect somaclonal variations, such as the polyploidy, genotype or donor plant, tissue culture source, culture duration, media components, and regeneration system [8,36]. In general, polyploid species are more variable in cell cultures compared with diploids, and different genomes develop different responses to stress due to cell cultivation [36]. At that, highly differentiated tissues of vegetative organs (roots, leaves, and stems) generally produce more variations than the axillary buds and shoot tips including the initial cells of meristems, and the use of undifferentiated tissues (pericycle, procambium, and cambium) as a source for tissue culture strongly reduces the possibility of variations [9,37]. *P. ginseng* was reported to be a natural polyploid, with two polyploidization events occurring during the evolution of this plant, allotetraploid origination, and chromosome numbers 2n = 24, 44, and 48 [14,38,39]. Moreover, different ancestral genomes can be developed at different rates [40], and different ginseng cultivars have no variation in the number of rDNA loci and which possess only one locus for each 5S and 45S rDNA [41].

Despite the abundance of literature on the somaclonal variation of plants, there is practically no information on the rDNA sequences of cultured cells. Therefore, the data obtained in this work on the variability of the primary and secondary structure of 18SrRNA in cultured ginseng cells are novel and of high priority. It has been suggested that a relatively high somaclonal instability can be associated with repetitive DNA due to the loss of control over mechanisms responsible for its high mutation rate and the release of instability mechanisms (amplification, translocation, chromosome breakage, and transposition) [41]. Apparently, this could also be true for the rDNA sequences considered as “sensors” of DNA damage and “shock absorbers” which prevent genomic damage [34]. rDNA damage can lead to the formation of an unusual secondary structure, which may cause the inhibition of DNA replication and the emergence of “hot-spot” recombination, inducing the translocation and change in the number of repeats. We revealed a number of mutations in the 18S rDNA, and although the main peaks of nucleotide diversity were localized in the non-conserved regions of the 18S rRNA secondary structure, the single-nucleotide substitutions were detected in presumably more functionally significant conserved regions as was also reported for cultivated *P. ginseng* plants [19].

For a long time, it was believed that through the mechanisms of concerted evolution the 18S rRNA genes are invariant within the genome and only a small variability may occur among individuals. It has since been found that in some cases this rule is not followed, and nucleotide variability can be substantial within the same genome [16,17,18,42]. The possible explanations for high variability in the rDNA sequences are polyploidy [43], interspecific hybridization [44], localization of nucleolar organizing regions on non-homologous chromosomes [43], and a higher mutational level in comparison with the rate of sequence homogenization [45].

Additionally, it has previously been shown that the 18S rRNA gene from ginseng plants already contains multiple nucleotide substitutions [19]. Thus, in *P. ginseng* cell cultures, the processes maintaining the 18S rRNA gene sequence homogeneity known as concerted evolution can fail to operate properly for at least two reasons: the high number of gene copies (resulting from polyploidization) and increased levels of nucleotide substitutions. The predisposition to nucleotide variability is inherited by the culture cells from their ancestors (tissue source).

The intragenomic mechanisms which may influence mutation fixation rates in gene families (particularly rDNA) comprise such processes as mutation, natural selection, genetic drift, and molecular drive (including gene conversion, recombination events, slippage, and transpositions) [46]. The level of nucleotide variability of the 18S rDNA sequences for cell lines (especially *1s*) appeared to be higher than that of the ginseng plant leaves [19] and the *P. ginseng actin*, *PAL*, *DDS*, and *SERK* genes [17]. However, as observed in this study, intragenomic nucleotide polymorphism for the 18S rDNA (on average 0.2–0.5%) was significantly lower than that (0.5–4.3%) of some other species of plants and animals [17,18], suggesting only a partial loss of the concerted evolution mechanisms in ginseng cell lines.

The whole-genome sequencing analysis performed for polyploid plants (*Arabidopsis* and maize) revealed single-nucleotide substitutions in protein-coding genes as the predominant type of mutations (presumably, to generate somaclones) [36]. Multiple-nucleotide substitutions are the most probable cause of both somaclonal variation and loss of the ability to produce biologically active substances during cultivation [7]. Herein, we found that this mutation pattern also prevailed in the 18 rDNA genes. The most frequent substitutions were A ↔ G and T ↔ C transitions, indicating that the native ginseng plant and cell lines of different cultivation times are exposed to similar mutation processes. At the same time, the *ts*/*tv* ratio was considerably lower in the long-cultivated cell lines, which could be the evidence of the very high level (comparable with interspecific) of genetic differentiation predicted for the long-cultivated cell lines [33]. Analogous transitional bias for the rDNA was also observed for parasitic plants, suggesting their highly specialized types of inter-organismal associations [47].

A large number of nucleotide substitutions could suggest an occurrence of pseudogenes among the cloned 18S rDNA gene sequences. Due to their high evolutionary rate, pseudogenes contribute to the extreme diversity of nuclear rDNA [18,44]. Pseudogenes of the small subunit ribosomal rRNA have been found in various organisms, ranging from bacteria [48] to primates. Although in theory “pseudogenization” is the most likely fate of the majority of extra gene copies, the most common fate for the set of duplicated genes is believed to be sub-functionalization, which is defined as the division of the function(s) originally performed by a single ancestral gene between its duplications [48]. In this respect, cell populations can be regarded as promising models for studying the evolutionary fate of duplicated genes. However, phylogenetic trees were not able to separate the analyzed gene sequences into two functionally different groups (i.e., genes and pseudogenes), as reported for the rDNA of other organisms [18,44]. This fact can be partially explained by the repeated stress which cells undergo because of regular passages, resulting in natural selection alterations on the one hand and an insufficiently long time of cell cultivation on the other.

In intraspecific comparison, the existence of alternative rRNA conformations for particular variable regions (usually not expected) could be evidence of both their high divergence and functional damage including pseudogenization. Consequently, data on the rRNA secondary structures could predict the possibility of disrupting the ribogenesis and protein synthesis in *P. ginseng* cell lines. The secondary structures for entire 18S rRNA were estimated to be quite conserved across the species studied, although those for variable regions differed significantly among themselves; however, no intraspecific variations have been reported until the present [24,49,50]. According to our data, only a few alternative variants have displayed conformations differing from canonical ones described for wheat [24]. A common alternative conformation for cell cultures (despite a high similarity with the plant by nucleotide sequences) was found for h17, which located (together with h16) at the mRNA entry site on the small ribosomal subunits. However, the secondary structure prediction of h17 is thought to be quite ambiguous [24]. At the same time, h17 was established to be an important phylogenetic marker; the loss of a nucleotide pair in this quite conserved region reliably distinguishes different taxonomic groups of nematodes [51].

In our study, no alternative conformations were obtained for highly conserve h18 (very similar in *Escherichia coli*, *Saccharomyces cerevisiae*, and humans), h33 (located on the beak of the small subunit and possessing variation only in protozoans), and h27. The h27 lied in the location of potentially large conformational dynamics and packed against the decoding center in h44 [52]. h27 may influence translation accuracy by affecting the conformation of the decoding region, and possesses a mutation that abolishes the ability of paromomycin to promote the readthrough of stop codons [53]. In the *E. coli* 16S rRNA, h27 was shown to have an innate tendency to exist in dynamic equilibrium between two conformations; a conformational switching model (between “accurate” and “error-prone” conformations) has non-been confirmed [52,53]. To some extent, this agrees with the existence of an additional secondary structure of h27 (very similar, with slightly higher-energy folding) for all alleles in all *P. ginseng* cell lines.

The most significant of the nucleotide differences among cell cultures which resulted in alterations of the secondary structures was localized in h8—that is, near the interaction site between two hypervariable regions, ES3 and ES6. Perhaps variations in h8 (both for interspecific and cell-line comparisons) are at least partially due to its adjacency with these regions. All the known 18S rRNA reconstructions support an interaction between ES3 and ES6, although its precise significance remains to be clarified. In *P. ginseng* plant and cell cultures, the ES6 interaction site for the majority of rRNA alleles consists of nine nucleotides (GUUGGCCUU), corresponding quite well with the consensus sequence (GUUGGUUUU) inferred for different groups of organisms [54,55]. Similar to some other species, the ES3 interaction site appeared to be more modified compared to the consensus sequence (AAAACCAAU) [56,57,58] and differed in cell cultures. As a result, the interaction sites of ES3 and ES6 in the ginseng plant can form seven complementary nucleotide pairs, while in cell cultures the number varied from six to eight (Figure 4C), which could be important under particular conditions.

rRNA sequence variations may alter the structure and function of the ribosome and its interplay with components of a cellular milieu, since the functions of many RNA types are defined by the formation of intramolecular base pairs [53,59,60]. There are numerous human diseases associated with rDNA dysfunction (ribosomopathies) caused by structural or functional abnormalities in the ribosomal components—including rDNA, which has a principal role in ribosomal function [32,53,61]. It is believed that, being a source of hypervariable genomic diversity, rDNA provides a mechanism for cellular homeostasis and adaptation, and ribosome heterogeneity is considered as ribosome-mediated control of gene regulation [61,62,63,64]. In addition, tissue-specific effects of ribosome dysfunction have also been demonstrated [63]. We found that some substitutions within hairpins affect the number of loops, and can make the secondary structure specific for a particular cell culture. The structural loops are acknowledged to be the most common functional motif of natural RNAs, often playing a major role in ensuring the specificity of RNA–RNA interactions [63,65]. Alterations in the number and size of symmetric interior loops lead to an alteration of the helical pitch, while asymmetric interior loops have been identified to fold into important tertiary structure formations (so-called structural motifs) [24].

Our results reveal significant alterations in the methylation profile for cell cultures different in age and tissue source ginseng. In plants, cytosine methylation is a common and flexible epigenetic regulator of gene expression that is important in normal development, in creating phenotypic diversity, and in modulating agronomical traits [32,55,56,59,66]. In plants, DNA methylation plays an important role in mechanisms of the abiotic and biotic stress response [67,68]. Genome-wide demethylation can cause abnormal development [69], and an abnormal methylation pattern can be lethal for the embryo [66,69]. Significant differences in cytosine methylation have been observed between different organs and different developmental phases of plants [57,66]. The cultivated ginseng plant maintains a high level of epigenetic diversity, and its cytosine methylation pattern differs from that of wild ginseng [58]. Recently, habitat-induced reciprocal transformation in the root phenotype of Oriental ginseng associated with DNA methylation was reported [70]. Assessments of the methylation status are useful for measuring the epigenetic “somaclonal” changes associated with passage through in vitro culture [71]. The methylation variation is present in in-vitro-regenerated agricultural and medicinal plants, and the frequency of these variants often increases with culture age [36]. Alterations in methylation patterns can be associated with methylase/demethylase activity; it was previously shown that the total demethylase (VaDem1 and VaDem2) expression in grape cell lines was lower than in the leaves of the native plant [72]. Although the biological functions of the rRNA modifications remain mainly unclear, alterations in the rRNA modifications occur during development, environmental changes, and disease, and dysregulation of methylation can affect ribosomal ligand binding and translation accuracy [73,74,75].

## 5. Conclusions

The data available in the literature indicate a high potential for the use of cell cultures; however, their implementation requires additional knowledge of physiology, metabolism, and genome of the plant cell, as well as new and non-standard approaches to solving the existing problems of cell engineering. Herein, during cell cultivation, we observed extensive age- and tissue-specific alterations, cytological and molecular, in the rDNA clusters of *P. ginseng* cell lines, which could affect the synthesis of highly specific biologically active ginsenosides. The divergent rRNA alleles identified in this study could provide additional gene expression regulation in *P. ginseng* cells in response to cultivation by means of the constitutions of heterogeneous ribosomes. The findings provide a framework for future exploration of how encoded rRNA variants can give rise to functionally heterogeneous ribosomes altering the pattern of intracellular synthesis. An understanding of the underlying mechanisms could help to prevent the loss of ability to produce biologically active substances in cell lines making cultivation more successful. The negative effects of long-term cultivation can be reduced by appropriate selection of the cell culture tissues, which is important for applied biotechnology. Advanced cell technologies based on the ginseng cell cultures will be able to provide humans with the necessary and effective heart drugs, high-quality cosmetics, and anti-aging remedies.

## Figures and Tables

**Table 1:** Statistics on mixoploidy in cell populations and the structure of interphase nuclei in the *Panax ginseng* cell lines.

Cell Lines	The Number of Nucleoli in Interphase Nuclei		The Range of Chromosome Number Variation	The Average Number of Chromosomes
	Macro Nucleoli (≥2 μm)	Micro Nucleoli (<2 μm)		
***9s***	**1.2 ± 0.1**	-	6–130	42.3 ± 2.4
***1s***	**5.6 ± 0.1**	4.3± 0.2	6–150	63.8 ± 2.8
***1o***	**6.3 ± 0.1**	5.5 ± 0.2	6–150	65.5 ± 2.5

**Table 2:** Nucleotide substitutions in the 18S rDNA clones of the *Panax ginseng* cell lines.

Cell Lines	*Ts*/*Tv*	Transitions				Transversions							
		A → G	T → C	G → A *	C → T *	A → T	A → C	T → A	T → G	G → T	C → G	G → C	C → A
*9s* (*n* = 20)	9.25	10 (24.4)	19 (46.3)	7 (17.1)	2 (4.8)	0	0	1 (2.4)	1 (2.4)	1 (2.4)	0	0	0
*1s* (*n* = 46)	2.60	49 (20.4)	39 (16.3)	23 (9.6)	55 (22.9)	43 (17.9)	7 (2.9)	6 (2.5)	1 (0.4)	5 (2.1)	1 (0.4)	4 (1.6)	7 (2.9)
*1o* (*n* = 46)	4.30	38 (25.0)	34 (22.4)	28 (18.4)	24 (15.8)	10 (6.6)	4 (2.6)	4 (2.6)	2 (1.3)	3 (2.0)	1 (0.7)	1 (0.7)	1 (0.7)
*Plant* (*n* = 30)	7.40	10 (23.8)	12 (28.6)	11 (26.2)	4 (9.5)	0	0	0	1 (2.3)	2 (4.7)	1 (2.3)	0	1 (2.3)

*n*: number of clones; * sites associated with cytosine methylation, *ts*/*tv*: ratio of transitions and transversions.

**Table 3:** GC content and characteristics of nucleotide substitutions in the 18S rDNA clones of *Panax ginseng* cell lines.

Sample	G + C, %	Met, %	Am, %	Met/Am	Substitutions per Gene		
					All Types	Methylation	Amination
*9s* (n = 20)	49.36	21.95	51.2	0.43	2.05	0.45	1.05
*1s* (n = 46)	49.31	32.50	19.1	1.70	5.20	1.70	1.00
*1o* (n = 46)	49.34	34.20	26.3	1.30	3.30	1.13	0.87
*Plant* (n = 30)	49.33	35.71	30.95	1.15	1.40	0.20	0.43

*Met*: nucleotide substitutions associated with cytosine methylation (C/T, G/A), *Am*: nucleotide substitutions associated with DNA amination (T/A, T/C, T/G).
